# Intravascular Imaging of Atherosclerosis by Using Engineered Nanoparticles

**DOI:** 10.3390/bios13030319

**Published:** 2023-02-24

**Authors:** Jiawen Li, Franco Centurion, Rouyan Chen, Zi Gu

**Affiliations:** 1 School of Electrical and Mechanical Engineering, University of Adelaide, Adelaide, SA 5005, Australia; 2 Australian Research Council Centre of Excellence for Nanoscale BioPhotonics, Adelaide, SA 5005, Australia; 3 Institute for Photonics and Advanced Sensing, The University of Adelaide, Adelaide, SA 5005, Australia; 4 School of Chemical Engineering, University of New South Wales, Sydney, NSW 2052, Australia; 5 Australian Centre for NanoMedicine (ACN), University of New South Wales, Sydney, NSW 2052, Australia; 6 UNSW RNA Institute, University of New South Wales, Sydney, NSW 2052, Australia

**Keywords:** engineered nanoparticles, atherosclerosis, intravascular imaging, fluorescence, photoacoustic imaging

## Abstract

Atherosclerosis is a leading cause of morbidity and mortality, and high-risk atherosclerotic plaques can result in myocardial infarction, stroke, and/or sudden death. Various imaging and sensing techniques (e.g., ultrasound, optical coherence tomography, fluorescence, photoacoustic) have been developed for scanning inside blood vessels to provide accurate detection of high-risk atherosclerotic plaques. Nanoparticles have been utilized in intravascular imaging to enable targeted detection of high-risk plaques, to enhance image contrast, and in some applications to also provide therapeutic functions of atherosclerosis. In this paper, we review the recent progress on developing nanoparticles for intravascular imaging of atherosclerosis. We discuss the basic nanoparticle design principles, imaging modalities and instrumentations, and common targets for atherosclerosis. The review is concluded and highlighted with discussions on challenges and opportunities for bringing nanoparticles into in vivo (pre)clinical intravascular applications.

## 1. Introduction

Atherosclerotic coronary artery disease (CAD) is the most common cause of death in middle- and high-income countries worldwide [1]. Each year, more than 20 million CAD patients worldwide experience acute coronary syndrome (ACS). Approximately 35% of these people die from ACS within a year [2].

To improve the management of CAD patients, we need to detect high-risk atherosclerotic plaques that are likely to cause ACS. Accurate detection of high-risk plaques could guide the use of more aggressive, and costly treatment strategies [3,4]. Novel systemic or “local” therapies [3] may be utilized in “vulnerable” patients or on high-risk plaques in “vulnerable” patients, respectively. Non-invasive imaging approaches are not able to accurately identify high-risk plaques, increasing the likelihood of myocardial infarction [5,6]. Non-invasive magnetic resonance imaging (MRI) can visualize heart walls and arterial systems but lacks high resolution to image coronary artery walls. Computed tomography (CT) has intrinsically low soft-tissue-contrast and high radiation exposure, limiting its applicability in coronary artery plaque risk evaluation. 

**Figure 1:**
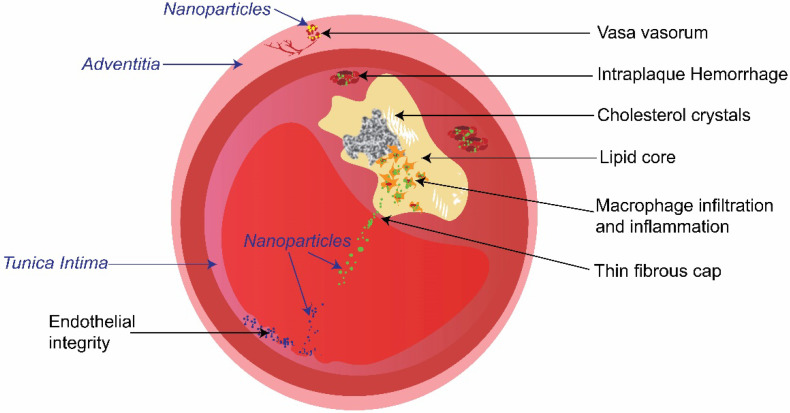
Schematic illustration of the cross-section of an atherosclerotic plaque in an artery. Key characteristics of the high-risk plaque (indicated by non-italic words in black), and typical intravascular targets of nanoparticles (nanoparticles targeting macrophages, endothelial cells, and vasa vasorum are represented by green, blue, and yellow dots respectively).

Plaque composition is of major importance for risk-assessment. Key characteristics of high-risk plaques (Figure 1) include morphological features (e.g., thin fibrous cap, cholesterol crystals, and accumulation of macrophages), and biochemical/functional characteristics (e.g., intraplaque hemorrhage, inflammation). To detect one or a few of these characteristics, intravascular imaging techniques such as intravascular ultrasound (IVUS) and optical coherence tomography (OCT) have been developed [7,8]. However, the intravascular imaging technologies currently utilized in hospitals (such as IVUS and OCT) either lack desirable resolution and/or molecular information, and thus suffer from significant drawbacks in detecting high-risk plaques [9,10]. As a result, the positive predictive values of these techniques are only between 18 and 41% [5,11], revealing their fundamental limitations. Novel intravascular imaging modalities and contrast agents (e.g., nanoparticles) have been studied to address these limitations [4,12].

**Figure 2:**
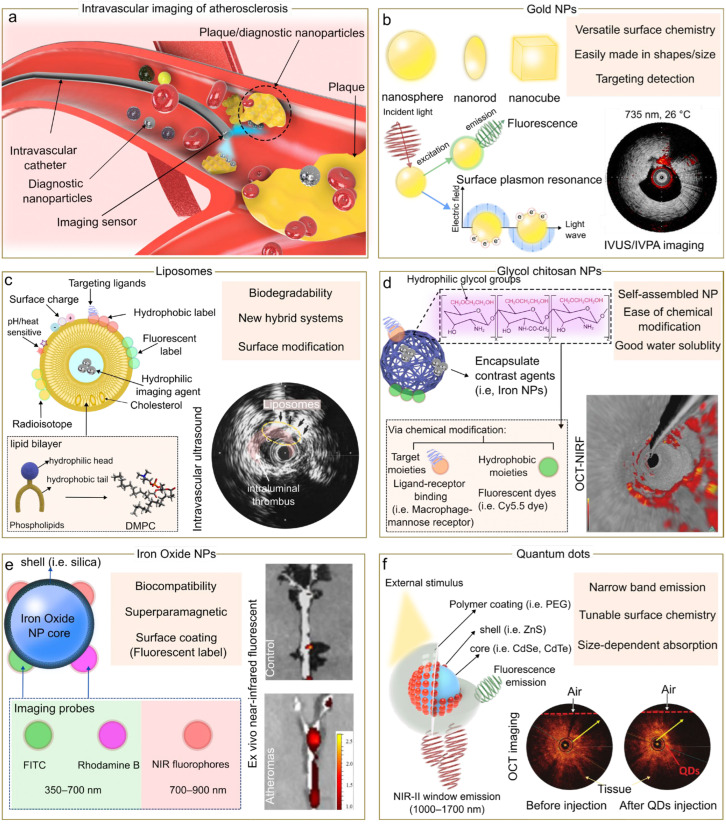
Nanomedicine approach for intravascular imaging of atherosclerosis. (**a**) Schematic representation of the use of nanoparticles for intravascular imaging. Comparison of nanoparticles (NP) probes for intravascular imaging (**b**–**f**) highlighting some benefits. Insets of (**b**,**d**,**e**) are reproduced from references [22,23,24] respectively, under the CC-BY license. Insets of (**c**,**f**) are reproduced from references [25,26] respectively, with permissions.

Engineered nanoparticles have emerged as a versatile tool that can integrate diagnostic screening and targeted therapy, enabling applications in various pathologies ranging from cancer to cardiovascular diseases [13,14]. Given the unique physical and chemical properties of nanoparticles, their functionality as nanotracers can be used to improve the image resolution and molecular contrast of clinically available and emerging intravascular imaging technologies [15,16,17]. Nanoparticles confer advantages in targeted delivery of imaging agents over conventional small molecule contrast agents [18]. Nanoparticles demonstrate a high surface area to volume ratio, which enables surface labelling with specific ligands at a high ratio of contrast agents to targeting ligands [19]. Nanoparticles are featured with modular structure that enables codelivery of imaging diagnostic and therapeutic agents on one platform, which are promising to be used as theranostic agents to simultaneously localize and treat diseases [20]. Furthermore, the nanoparticle formulation can be controlled to be designed in different sizes and geometries, resulting in an enhancement of their optical properties [21]. Despite the current progress in nanoparticle-based imaging, intravascular imaging using nanoparticles (Figure 2a) is still in its infancy. The development and future translation of nanoagents for intravascular imaging are forecasted to improve the prognosis and therapy selection.

To facilitate future development of nanoparticles for intravascular imaging, we describe engineered nanoparticles and their applications in intravascular imaging (Section 2), typical intravascular imaging techniques (Section 3), and commonly used targeting mechanisms for high-risk plaque detection (Section 4) in this review, and also discuss exciting future directions, challenges, and opportunities toward clinical and preclinical applications of nanoparticles for intravascular imaging. 

## 2. Nanoparticle Types for Intravascular Imaging

### 2.1. Gold Nanoparticles

Gold nanoparticles (Figure 2b) have been widely used in a range of biomedical applications including drug delivery, photothermal therapy, and imaging [27,28]. This broadspectrum of applications is mainly due to their unique optical and electronic properties. One of the main optical properties of gold nanoparticles is based on localized surface plasmon resonance which leads to generating a powerful electromagnetic field on metal nanoparticle surface, enhancing absorption and scattering in the visible and near-infrared region [29,30]. For example, gold nanoparticles can be used as exogenous absorbers and be introduced to the plaque to enhance intravascular photoacoustic imaging (IVPA) at the molecular or cellular level. It was also demonstrated that gold nanoparticles can be aggregated in the macrophages producing a plasmon resonance coupling of nanoparticles inside the cells. As a result, a red-shift effect was observed in the optical spectra, allowing IVPA imaging at 700 nm [31]. 

Given that the optical, magnetic, catalytic, and electrical attributes of gold nanoparticles are influenced by the size and shape, their design has been studied in a variety of forms ranging from nanospheres, nanorods, nanoshells, and even nanocages. For example, gold nanorods have stronger and tunable absorption properties including enhanced photoluminescence and surface-enhanced Raman scattering (SERS) as compared to nanospheres. This phenomenon is explained by the split of the plasmon band in the nanorod shape which originates a longitudinal and transverse plasmon band [32]. Furthermore, the miniaturization of gold nanorods to the scale of 8 nm by 49 nm has been demonstrated to impact the optical properties, being three times more thermally stable and generating 3.5 times enhanced photoacoustic signal than their larger counterparts [33].

In addition, gold nanoparticles are also known to exhibit SERS properties. As such, gold nanoparticles and Raman-active molecules were encapsulated in a silica shell to be further functionalized with intercellular adhesion molecule 1- (ICAM-1-) specific monoclonal antibodies. As a result, the resulting gold nanoparticle composite displayed higher sensitivity detection of ICAM-1 over conventional fluorophore–antibody conjugates. On top of that, it also provides an enhanced spectral definition, and depth resolution in in vitro, ex vivo, and in vivo models [34]. Another recent strategy used in clinical cardiovascular medicine is intravascular optical coherence tomography imaging, in which gold nanoshells can trigger the backscattered signal produced by individual cells at a specific OCT laser wavelength of 1300 nm [35]. 

### 2.2. Liposomes

Liposomes (Figure 2c) are spherical vesicles (50–200 nm) that enclose an aqueous core and a lipid bilayer membrane [36]. Due to their intrinsic hydrophilic and hydrophobic nature, liposomes have been extensively used to encapsulate a variety of therapeutic agents including proteins, nucleic acid, drugs, and diagnostic agents [37]. In cardiovascular research, earlier studies showed that liposomal particles can be accumulated in infarcted myocardium [38], and more recent studies suggest that liposome particles with sizes between 70 to 110 nm are associated with more capabilities to penetrate capillaries and be accumulated in the ischemic myocardium [39].

Considering the broad diversity of lipid molecules, the composition and functional properties of liposomes such as particle size, rigidity, fluidity, stability, and electrical charge can be tuned [40]. Conventionally, liposomes are synthesized from natural (i.e., phosphatidylcholine, phosphatidylethanolamine, phosphatidylinositol), or synthetic phospholipids (i.e., 1,2-dipalmitoyl-sn-glycero-3-phosphocholine, 1,2-dimyristoyl-sn-glycero-3-phosphocholine (DMPC)), and the incorporation of cholesterol in their formulations has shown to improve the liposome fluidity, stability, and drug release. Furthermore, the liposome surface can also be modified with sheath molecules (e.g., polyethylene glycol) to increase the circulation time. For example, gadolinium-loaded liposome conjugated with a polyethylene glycol (PEG) coating has been shown to provide an extended intravascular circulatory half-life of up to 18 h. This effect is mainly due to the PEG coating which can act as a shield against opsonins and macrophages of the reticuloendothelial system [41]. In cardiovascular imaging, a specific fluorescent probe (peptide-ICG2) that is activated in the presence of cathepsin B was encapsulated in phosphatidylserine-conjugating liposomes. The resulting fluorescence imaging probe was sensitive to be activated when the activated macrophages from atherosclerotic areas were expressing cathepsin B. As a result, the probe emitted a near infrared (NIR) fluorescence signal for the detection of atherosclerotic plaques [42,43]. 

### 2.3. Glycol Chitosan Nanoparticles

Glycol chitosan (GC) nanoparticles (Figure 2d) have been established as promising nanocarriers for tumor targeting. Over the past decades, the encapsulation of contrast agents using glycol chitosan nanoparticles has been highlighted by their accumulation at the tumor areas in animal models [44].

Glycol chitosan is a multifunctional water-soluble polymer containing hydrophilic glycol groups. In addition, their hydrophilic nature can be easily transformed into a hydrophobic entity via the conjugation of their amine group. As such, this chemical versatility is widely explored to produce multiple drug delivery carriers to target tumor sites [44]. 

In cardiovascular medicine, the development of hydrophobically modified glycol chitosan (HGC) has been studied for imaging atherosclerosis plaque. In particular, HGC nanoparticles were conjugatd with an atherosclerotic plaque-selective peptide (AP peptide) to bind to the atherosclerosis plaque. As a result, the AP-peptide-conjugated HGC nanoparticles bound specifically to cytokine (TNF-α)-activated bovine aortic endothelial cells. Fluorescence imaging was achieved via labelling a NIR fluorophore (i.e., Cyanine, Cy5.5) [45]. As such, GC nanoparticles can serve as an anchor point for the incorporation of NIR fluorescent dyes [45] and even radioactive agents [46]. 

Based on the physical and chemical properties of GC nanoparticles, the molecular weight of the GC nanoparticles was shown to affect the tumor-targeting efficacy [47], identifying that the highest molecular weight (~250 kda) of GC nanoparticles display prolonged circulation time compared to the smaller ones (~20 kda). Other studies showed that HGC nanoparticles modified with N-acetyl histidine can modify their biodistribution, showing a higher concentration of GC nanoparticles in tumors when containing 7.8% than 3.3% of N-acetyl histidine [48]. Moreover, a key factor in the design of GC nanoparticles is deformability. Nanoparticles with high deformability can be desired to escape from physiological processes such as splenic filtration. The degree of substitution in GC nanoparticles has been associated with significant changes in stability and deformability. For example, when the GC nanoparticles were produced with a 23% of substitution, the deformability and stability were found to be optimal. However, when the degree of substitution was increased to 35%, the particles were not able to pass through the filter despite their increased stability [49].

### 2.4. Iron Oxide Nanoparticles

Iron oxide nanoparticles (IONPs, Figure 2e), i.e., magnetite (Fe_3_O_4_) or maghemite (γ-Fe_2_O_3_), nanoparticles have been used for intravascular imaging.

IONPs have been used as MRI contrast agents and approved by Food and Drug Administration (FDA) [50]. In cardiovascular research, ultrasmall superparamagnetic iron oxide conjugated with vascular cell adhesion molecule-1 and apoptotic cell-targeted peptides were used to detect vulnerable plaques [51]. In another work, ultrasmall superparamagnetic particles of iron oxide (USPIO) with a diameter of 5 to 50 nm have been developed to image myocardial and vascular inflammation. This type of IONPs can be uptaken by monocytes and macrophages at the site of inflammation [52]. Unlike larger IONPs, USPIO have been demonstrated to be more suitable for MRI tracking due to higher cellular uptake by nonphagocytic cells (e.g., mesenchymal stromal cells) [53]. Furthermore, the small size of USPIO leads to escape from immediate phagocytosis processes, resulting in an extended half-life of up to 36 h in comparison with 2 h for larger IONPs in humans [54]. In addition, antibody-conjugated superparamagnetic iron oxide nanoparticles showed a high accumulation in cardiac muscles, and has potentials to be a cardiac precursor label to segregate cells from cardiac/non cardiac origins and traced by MRI [55].

Moreover, the success of IONPs internalization into non-phagocytic cells also depends on surface chemistry. For instance, dextran-coated IONPs were used to attach a fluorescent peptide to enhance intracellular magnetic labeling [56], and used as well for multimodality imaging of atherosclerotic plaques [57]. IONPs are typically complexed with organic fluorochromes such as Cy5.5 dye, rhodamine, and fluorescein isothiocyanate, where the NP core is surrounded by coatings consisting of silica, lipids, and polymers. Furthermore, the conjugated probes should be able to avoid photobleaching, display separated emission-absorption spectra, and have long-term stability [58]. The benefits of using a coating on IONPs are also essential to increase their hydrophilicity, prevent aggregation, reduce binding to plasma proteins, and improve conjugation with targeting ligands [54]. 

### 2.5. Quantum Dots

Quantum dots (QDs, Figure 2f) are semiconductor particles with unique optical and electronic properties that have attracted great attention in biological imaging [59]. The quantum confinement feature displayed by QDs enables to have broad absorption spectra with a narrow emission band, serving as a versatile fluorescent probe for imaging and detection of multiple molecular targets [60]. QDs are typically in the range of 2–10 nm in size and are generally classified based on their composition in core-type, core-shell, and alloyed quantum dots. Core-type QDs are mainly based on a single component such as chalcogenides of metals (i.e., CdTe, PbS, ZnTe); meanwhile, core-shell QDs which are designed to improve photoluminescence properties are based on a growing shell (a wide-bandgap material) onto the core of the semiconductor. On the other hand, alloyed quantum dots are prepared by incorporating multiple components in a gradient internal structure (e.g., cadmium zinc selenide) which allows the tuning of the optical properties without changing the QDs size [61]. 

Among the optical properties, the emission spectra of QDs can be easily tuned by adjusting the particle size and composition using a set of different surface coatings. As such, QDs can display a variety of wavelengths ranging from ultraviolet to infrared. Besides that, a singular advantage of QDs is photostability which has been shown to reach repeated fluorescence cycles for hours in comparison with only a few minutes for conventional organic fluorophores [62].

For in vivo imaging using QDs, the benefit is arising in the engineering of QDs to fluoresce in the NIR optical region (700–1700 nm) where the tissue absorbance and scatter are lower. As such, Ag_2_S nanodots have been studied as contrast agents for the visualization of myocardial tissue in the second biological window (NIR-II). The surface modification of QDs with angiotensin II peptide enabled a high luminescence feature with a specific affinity to ischemic areas [63]. In another study, infrared-emitting QDs were used as a multimodal intracoronary imaging agent (OCT+NIR) under single-line laser excitation. This approach demonstrates that the excitation of QDs produced backscattered radiation which was strong enough to be detected by OCT, and simultaneously the absorbed photons were able to be redirected and measured by fluorescence imaging [26]. 

## 3. Intravascular Imaging Modalities

In this section, an overview of various imaging modalities and related nanoparticle studies are provided.

### 3.1. IVUS

IVUS has been clinically available for more than three decades [64,65]. IVUS creates three-dimensional (3D) images of the artery wall and plaques by rotating an ultrasound sensor and obtaining the reflected echo. As the contrast of IVUS is based on acoustic scattering and absorption of the biological tissue, which is not specific to any chemical composition, IVUS alone is not able to provide molecular contrast. Nanoparticles have been used as an image contrast agent for ultrasound to enable molecular imaging capability with IVUS [66,67]. Unlike most existing ultrasound contrast agents, which tend to be larger than 1 µm in size, nanoparticles are small enough to penetrate the arterial wall (even including adventitia via vasa vasorum) [66]. In particular, echogenic liposomes have been used to conjugate antibodies against adhesion molecules, tissue factors, and thrombotic markers to image the development of plaques [66,67]. 

### 3.2. OCT

Intravascular OCT has been used in patients since 2008 [68]. OCT creates 3D images of plaques with a penetration depth of no larger than 1 mm, which is lower than clinical available IVUS (5–10 mm). OCT enables a higher spatial resolution (5–30 µm) compared to ultrasound (50–300 µm). Although there is only a few studies utilizing nanoparticles in intravascular OCT, they have been used as image contrast agents, either to improve OCT image quality by increasing scattering [35], or to enable molecular imaging capability with OCT [26].

### 3.3. Fluorescence

Fluorescence is a commonly used molecular imaging method with a high spatial resolution (0.3–300 µm). To enable fluorescence imaging inside the blood vessel, intravascular fluorescence catheter was developed and reported in 2008 [69] and two-dimensional (2D) intravascular fluorescence was achieved in 2011 [70]. Despite these research catheters, no clinical intravascular fluorescence device has been launched to date. This is partially because fluorescence does not provide anatomical imaging capability, which is needed for plaque characteristics. Several groups have been developing hybrid OCT and fluorescence intravascular imaging catheters to provide molecular and anatomical imaging functions simultaneously [71,72,73]. 

A wide range of fluorescent dyes are commercially available, and some are FDA-approved for various clinical indications (e.g., 5-aminolevulinic acid, fluorescein and indocyanine green [74]). On the other hand, a large number of nanoparticles have been developed to enable targeted fluorescence-based cardiovascular imaging [75,76]. In addition to the advantages summarized in the introduction, nanoparticles also can overcome some limitations of common fluorescence systems, e.g., loading a greater number of fluorescent dyes into the high-risk lesion by active or passive strategies, preventing quenching, and reducing photobleaching [72]. Nanoparticles that target fibrin [71], macrophages [23], endothelial function [77], and intraplaque hemorrhage [78] have been used with intravascular fluorescence devices.

### 3.4. Photoacoustic Imaging

Photoacoustic imaging is an emerging molecular imaging technique [79]. It provides deeper imaging depth than pure optical imaging method (e.g., fluorescence) by utilizing ultrasound detection through the photoacoustic effect. As it only provides molecular contrast, similar to fluorescence, photoacoustic imaging often pairs with ultrasound to enable complementary anatomical imaging capability. Research groups and companies have been actively developing intravascular photoacoustic (IVPA) devices [80,81,82,83,84], but there is no clinical available IVPA yet. Nanoparticles were used for IVPA as targeting agents [31] soon after the invention of IVPA [80]. They have been used to target macrophages [31] and enzymes (e.g., matrix metalloproteinases) [85], etc. 

## 4. Targeting Mechanisms of Nanoparticles for Intravascular Imaging

As discussed in the previous sections, nanoparticles are to provide targeted imaging of atherosclerosis. In this section, we summarize common targeting mechanisms for the detection of high-risk plaques characteristics, including macrophages (Section 4.1), lack of endothelial integrity (Section 4.2), MMP-2 enzyme (Section 4.3), and neovessels (Section 4.4). 

### 4.1. Macrophages Targeting

Macrophages (Figure 1) play a critical role in multiple stages of the development of atherosclerosis. For example, infiltration of monocyte-derived macrophages into the sub-endothelial space of arteries drives the inflammation process of atherosclerosis formation. This is followed by the uptake of oxidized low-density lipoproteins by the macrophages and thus leading to the formation of foam cells and eventually forming the lipid-laden core of the atherosclerotic plaque [86]. 

The macrophage mannose receptor (MMR, Cluster of Differentiation CD206) is one of the key macrophage receptors for targeting a subgroup of macrophages that are abundant in unstable plaques and are present far from the lipid core, mainly in the fibrous cap of atherosclerotic plaques [23]. MMR is highly effective in mediating endocytosis and phagocytosis, which suggests that MMR is a potential target for a more specific intake of nanoparticles for atherosclerotic plaque imaging and diagnosis. In Kim and colleagues’ fluorescence imaging and OCT study, a Cy7 labeled, PEG, mannose receptor binding ligand attached, glycol chitosan-based nanoparticle was investigated in an in vivo high-risk plaque rabbit model. The study results signify that highly intense fluorescence signals colocalized with the presence of macrophages in the luminal area, which was validated through the immunohistochemical staining RAM-11 [23]. Furthermore, specific binding to MMRs also provides a quantification of macrophage contents [23]. This group further explored the effect of injecting the nanoprobe in intraplaque hemorrhage (IPH) induced in rabbit aortas. This study specifically investigated a distinct macrophage phenotype that expresses CD163 and CD206, differentiated in a hemoglobin rich environment [78]. Results suggest that stronger fluorescence signals were observed in sections with IPH formation, and this was validated by the comparison with CD206 staining. The group also took their nanoparticle further, synthesized into a nanodrug (mannose-polyethylene glycol-glycol chitosan-deoxycholic acid-cyanine 7-lobeglitazone, MMR-Lobe-Cy) by loading a peroxisome proliferator-activated receptor γ (PPARγ) agonist, lobeglitazone, which can identify inflammatory activity and deliver the drug that activates the cascade behavior that inhibits the Toll-like receptor 4 (TLR4)/ nuclear factor-κB (NF-κB) pathway, thus reducing inflammation. The study group administered with the MMR-Lobe-Cy had notably decreased inflammatory fluorescence signals compared to the saline control [72]. Furthermore, it resulted in a phenotypical change in plaque type, from an inflamed plaque to a more stable phenotype [72]. 

### 4.2. Endothelial Cell Targeting

Endothelial cells (Figure 1) form the lining of blood vessels and are also critical players in inflammatory responses in atherosclerotic plaques. The endothelial cells become activated by turbulent blood flow, deposition of lipids in the blood vessel, and exposure to inflammatory biomarkers such as interleukin-1β [87]. ICAM-1 is an adhesion molecule that is expressed by endothelial cells, and in atherosclerotic plaques, the expression of ICAM-1 is elevated. ICAM-1 assists in the recruitment of inflammatory cells, namely leukocytes and monocytes [88]. Therefore, it can be a useful target for imaging atherosclerotic plaques, as demonstrated both non-invasively [89] and invasively [66]. It is worth to note that the discussion here is limited to invasive imaging as the focus of this review is intravascular imaging, which provides higher spatial resolution than non-invasive imaging techniques. In Kee and colleagues’ IVUS study, ICAM-1 has been chosen to be the target molecule, by attaching an anti-ICAM-1 antibody to liposomes [66]. This facilitated the adhesion to the early stage atherosclerotic plaques. The pre-treatment of nitric oxide-echogenic liposome (ELIP) dilated the vessel and allowed the anti-ICAM-1 ELIP to penetrate subendothelial layers, thus showing an enhanced ultrasound echogenicity. 

Another method of targeting endothelial cells is through binding to ανβ3 integrin. In activated endothelial cells, the integrin ανβ3 is strongly upregulated [90]. This integrin is responsible for angiogenesis and can specifically bind to RGD peptide (Arginylglycylaspartic acid). Lin et al. synthesized a human serum albumin (HSA)-based nanoparticle which was modified by the RGDfk peptide and attached with the indocyanine green (ICG), named IHR-NPs, for their IVUS+IVPA study [90]. Histological staining of vWF, αvβ3, and CD31 indicated the formation of neovascularture, and this was supported by the high photoacoustic signals in intravascular imaging. 

### 4.3. Matrix Metalloproteinase-2 Enzyme Targeting

Matrix metalloproteinase-2 (MMP-2) enzyme is present in various pathophysiological settings. At early stages of atherosclerosis, MMP-2 can degrade the basement membrane of endothelial cells and allows the low-density lipoproteins to penetrate the tunica intima (Figure 1). Matrix metalloproteinase plays an important role in the rupture of unstable plaques as it can break down the extracellular matrix and degrade the fibrous cap integrity, thus leading to plaque rupture. Hence, the attachment of MMP-2 antibodies leads to specific binding to areas of MMP-2 secretion, targeting more unstable plaque phenotypes. Qin et al. reported that a high MMP-2 area corresponds with a high photoacoustic signal intensity, and this was validated with immunofluorescence imaging. Through MMP-2 antibody conjugation, the gold nanorods can be delivered to the inflammation site, which enables its IVPA imaging capability [85]. 

### 4.4. Passive Targeting

Passive targeting is another strategy to deliver nanoparticles to atherosclerotic plaques for intravascular imaging. The nanoparticles can enter the plaque through the enhanced permeability and retention effect due to the openings in the blood vessel lining [91].

Yeager and colleagues suggested that the dysfunctional endothelial or fibrous cap implies that their PEG-gold nanorod can leak into the plaque due to the “discontinuous luminal boundaries” in their IVPA study [92]. In addition, a heterogeneous deposition was reported in a fluorescence study conducted by Stein-Merlob et al. [77]. The study suggested that the deposition of their ultrasmall superparamagnetic iron oxide nanoparticle, named CLIO-Cy-Am7, was primarily found in the first 100 µm of the atherosclerotic plaque. This is due to the impairment of the endothelial barrier function and was further supported by the correlation between Evans blue deposition in the intima layer of the blood vessel and the CLIO-CyAm7 penetration. The heterogeneity of the nanoparticle deposition suggests that the altered endothelial permeability influences the nanoparticle deposition [77]. The study concluded that the USPIO nanoparticles not only accumulated in the macrophages but also were found in inflammatory smooth muscle cells and intimal endothelial cells. The authors suggested that the inflammatory smooth muscle cells can phagocytose the CLIO-CyAm7 as it is similar in size to low-density lipoproteins [77]. Similar studies also suggested that nanoparticles could be phagocytosed by macrophages, as evidenced shown by immunohistochemistry RAM-11 staining of macrophages [92].

Another method of passive binding to the atherosclerotic plaque is through the neovessels formed at the adventitia, namely the vasa vasorum (see Figure 1). A study by Lobatto et al. indicated that with the injection of fluorescent Cy5.5 loaded liposome, nanoparticle presence was found in both luminal side and the vasa vasorum at 6 h. By 24 h, the nanoparticle was found to be dispersed throughout the plaque [93].

## 5. Challenges and Opportunities

Despite recent studies, as reviewed in the previous sections, the development of nanoparticles for intravascular imaging remains in its infancy. This section discusses the challenges of utilizing nanoparticles for preclinical and clinical applications, potential strategies to address the challenges, and exciting opportunities by leveraging latest advances in chemical engineering and photonics.

### 5.1. Challenges in Nanomedicine 

#### 5.1.1. Toxicity 

A critical challenge for the clinical application of nanoparticles is toxicity. For example, studies have shown that superparamagnetic iron oxide nanoparticles may cause myocardial injury [94]. The most time- and cost- efficient way to test toxicity is 2D in vitro assay [95]. Although it is more well-controlled in terms of experiment conditions, it neglects the complexity of the 3D body. Thus, animal testing and novel 3D in vitro assays [96] are also utilized to test the toxicity of a nanoparticle. Size and surface charge are critical factors that affect the toxicity of a nanoparticle. A study by J. Matuszak et al. has particularly investigated the toxicity of nanoparticles for intravascular applications [97]. This study utilized the published nanoparticles (liposomes, lipid, polymetric and iron oxide nanoparticles). The majority of tested nanoparticles (up to 100 μg/mL in static) did not cause significant negative effects (on cell viability or adherence) to endothelial cells, i.e., the first-contact vascular cells for intravascular applications. This study also indicated that smaller size lipid nanoparticles were less tolerated by endothelial cells, probably due to the effect of higher surface concentration and the small size facilitating the uptake. In addition to 2D in vitro toxicity test, this study also investigated the in vivo responses of liposomes at two different concentrations in domestic pigs, which further validated the low immunogenicity of the nanoparticles. 

#### 5.1.2. Batch-to-Batch Variation and Industry-Scale Manufacturing

A fundamental barrier to the clinical translation of nanomedicine is its intrinsic difficulties in batch-to-batch reproducibility (in terms of size distribution, coating, surface charge, etc.,) [98]. It has been demonstrated that batch-to-batch variation may lead to adverse effects (e.g., oxidative damage of proteins or DNA) [99]. Most studies in nanoparticles for intravascular imaging (summarized in Table 1) are limited to small scale, lab environment. Challenges with industry-scale manufacturing has been widely experienced [100]. Microfluidics with capabilities to control nanoparticles’ properties has been developed to make nanomedicine for drug delivery [101,102]. Rapid scaling up has been demonstrated by utilizing this microfluidic technique [102] and similar techniques may enable consistent manufacturing process of nanoparticles for imaging.

#### 5.1.3. Regulation

Finally, yet importantly, complicated regulatory processes cause additional challenges for the clinical translation of nanoparticles. A novel nanoparticle needs to get approved by the regulatory agent of the specific market before it can be used in that market/country (e.g., FDA for USA, Conformité Européenne, CE for Europe, China FDA for China, Therapeutic Goods Administration, TGA for Australia). Some nanoparticles can be classified as medicine in one country but a medical device in another [103]. The class of the device (e.g., Class I, Class IIa, Class IIb, Class III in the FDA guidelines), which is related to the risk of the product and determines how complicated its regulatory process is, can also vary between countries [103]. Accordingly, the safety and efficacy data obtained for one country/market is not always useful for the regulatory approval of another country [95]. Furthermore, the batch-to-batch variance and challenges in consistent large-scale manufacturing, as discussed in 5.1.2, makes it more difficult for nanoparticles to get approved than a small molecule medicine [98].

Before obtaining regulatory approval for clinical use, one potential application of nanoparticles is to utilize its active/targeted labelling capability to compare side-by-side with label free imaging technologies in animal models in vivo. This kind of studies can help us better understand plaque destabilization process and potentially identify the most suitable imaging modality for each particular application [16] (e.g., detection of macrophages [16] or endothelial cells).

### 5.2. Specific Challenges or Opportunities for Intravascular Imaging

#### 5.2.1. Increasing Accumulation of Nanoparticles at the Target Site

Efficient targeting and accumulation of nanoparticles at the atherosclerotic plaque is a key to nanoparticle-enhanced intravascular imaging. After intravenous administration, the nanoparticles need to overcome immune recognition and penetrate through the openings in the impaired vascular endothelium or in those of neovessels to reach the atherosclerotic plaques. Although a variety of targeting strategies have been developed, the accumulation remains suboptimal [104]. In recent years, biomimetic delivery by cloaking nanoparticles in naturally derived cell membranes have attracted significant interests in nanomedicine, which could be promising to amplify homing of nanoparticles to the plaques.

#### 5.2.2. Targeting Multiple Atherosclerotic Characteristics Simultaneously

As shown in Figure 1, there are multiple biochemical/functional characteristics (e.g., intraplaque hemorrhage, inflammation, lipid core) which are associated with the risk of a plaque. To target multiple characteristics at the same time, nanoparticles can be engineered with distinct absorption/emission peaks or fluorescence lifetime [105]. For example, with spectroscopic photoacoustic, multiple targets can be detected simultaneously by utilizing multiple nanoparticles that have distinct absorption peaks [27]. Dual/multiple targeting by using two or more ligands [106] to target different receptors on a cell or target hierarchical biological barriers could also increase the accumulation of nanoparticles at the atherosclerotic lesion site for next-generation intravascular imaging.

#### 5.2.3. Visualizing Physiological Processes

Distinct metabolism at the atherosclerotic lesion results in various physiological characteristics that distinguish the lesion from normal tissues and thus provide biological targets and stimulus for selective molecular imaging. With the progress in development of various stimuli-responsive imaging probes, quantitatively visualizing the physiological hallmarks and processes in vivo remains challenging, given that a substantial number of engineered imaging probes can only detect the target in vitro or provide anatomical information of the lesion in vivo. Recent advances in chemical construction of smart responsive nanoparticles enable a new horizon for quantitatively monitoring the biological abnormalities in vivo, including pH variation, enzyme overexpression, reactive oxygen species elevation and hypoxia [107], which is promising to be leveraged to intravascular imaging of atherosclerosis.

## 6. Conclusions

In summary, nanoparticles provide great hope for targeted imaging of high-risk atherosclerotic plaques (e.g., via targeting macrophages, endothelial cells, enzymes and neovessels). Gold nanoparticles, liposomes, glycol chitosan nanoparticles, iron oxide nanoparticles, and quantum dots have already been utilized for imaging intravascularly. Despite challenges for their clinical translation, engineered nanoparticles have played an important role in preclinical studies and will continue to provide new opportunities to advance intravascular imaging of atherosclerosis.

## Figures and Tables

**Table 1:** Works published to date on the use of nanoparticles for intravascular imaging.

Imaging Modality	Nanoparticle Type	Wavelengths	Passive/Active	Application	Ref
Fluorescence (and OCT)	Cross-linked iron oxide (CLIO)	Excitation: 750 nm (Cy7)	Active: a stent coated with fibrin-targeted nanoparticle	Fibrin deposition on stent indicative of stent thrombosis	[71]
Fluorescence (and OCT)	Glycol chitosan nanoparticles	Excitation: 633 nm (Cy5.5)	Active, targeting macrophage mannose receptors	Direct estimation of plaque macrophage contents	[23]
Fluorescence (and IVUS)	Iron oxide nanoparticles	Excitation: 750 nm	Passive	Endothelial functional integrity	[77]
Fluorescence (and OCT)	Glycol chitosan nanoparticles	Excitation: 750 nm and emission 773 nm (Cy7)	Active, targeting macrophage mannose receptors	Intraplaque hemorrhage	[78]
Fluorescence (and OCT)	Glycol chitosan nanoparticles	Excitation: 750 nm and emission 773 nm (Cy7)	Active, targeting macrophage mannose receptors	Macrophage targeted theranostic strategy	[72]
OCT	Gold nanoshells	~1300 nm	Passive	Enhancing the backscattered signal of individual cells	[35]
OCT and fluorescence	Quantum dots	~1300 nm	Passive	Enhancing the backscattered signal of individual cells and also utilizing OCT laser to excite the quantum dots	[26]
IVUS	Liposome	N/A	Active	An echogenic liposome (ELIP) consisting of various polar lipids, including one containing rhodamine that gains echogenicity through encapsulation of air.	[66]
IVPA	Gold nanoparticles	Excitation: 680 nm	Passive	Photoacoustic signal from nanoparticles internalized by macrophages is very strong due to the plasmon resonance coupling effect.	[31]
IVPA	Gold nanorods	Spectroscopic system (730 nm–830 nm) and single wavelength system (at 750 nm)	Passive	Detection of compromised luminal endothelium and acute inflammation in the presence of luminal blood	[92]
IVPA	Silica-coated Gold nanorods (SiO_2_AuNR)	Imaging performed at SiO_2_AuNR absorption peak of 735 nm; Laser heating: 808 nm	Passive	Monitoring local temperature rise during selective laser heating of SiO_2_AuNR	[22]
IVPA	Gold nanorod	695 nm, as the nanorod used has a resonance absorption peak of ~695 nm	Active, by gold nanorod conjugated with an antibody	Matrix metalloproteinases	[85]

## Data Availability

No new data were created.
